# Reassessment of the Psychometric Characteristics and Factor Structure of the ‘Perceived Stress Questionnaire’ (PSQ): Analysis in a Sample of Dental Students

**DOI:** 10.1371/journal.pone.0087071

**Published:** 2014-01-23

**Authors:** Jesús Montero-Marin, Marcelo Marcos Piva Demarzo, Joao Paulo Pereira, Marina Olea, Javier García-Campayo

**Affiliations:** 1 Faculty of Health and Sport Sciences, University of Zaragoza, Zaragoza, Spain; 2 Department of Preventive Medicine, Universidade Federal de São Paulo, São Paulo, Brasil; 3 Departament of Psychology, Instituto Superior da Maia, Castelo da Maia, Portugal; 4 Miguel Servet University Hospital, Zaragoza, Spain; 5 Primary Care Prevention and Health Promotion Research Network (RedIAPP), Zaragoza, Spain; Catholic University of Sacred Heart of Rome, Italy

## Abstract

**Background:**

The training to become a dentist can create psychological distress. The present study evaluates the structure of the ‘Perceived Stress Questionnaire’ (PSQ), its internal consistency model and interrelatedness with burnout, anxiety, depression and resilience among dental students.

**Methods:**

The study employed a cross-sectional design. A sample of Spanish dental students (n = 314) completed the PSQ, the ‘Goldberg Anxiety and Depression Scale’ (GADS), ‘Connor-Davidson Resilience Scale’ (10-item CD-RISC) and ‘Maslach Burnout Inventory-Student Survey’ (MBI-SS). The structure was estimated using Parallel Analysis from polychoric correlations. Unweighted Least Squares was the method for factor extraction, using the Item Response Theory to evaluate the discriminative power of items. Internal consistency was assessed by squaring the correlation between the latent true variable and the observed variable. The relationships between the PSQ and the other constructs were analysed using Spearman’s coefficient.

**Results:**

The results showed a PSQ structure through two sub-factors (‘frustration’ and ‘tenseness’) with regard to one general factor (‘perceived stress’). Items that did not satisfy discriminative capacity were rejected. The model fit were acceptable (GFI = 0.98; RSMR = 0.06; AGFI = 0.98; NFI = 0.98; RFI = 0.98). All the factors showed adequate internal consistency as measured by the congeneric model (≥0.91). High and significant associations were observed between perceived stress and burnout, anxiety, depression and resilience.

**Conclusions:**

The PSQ showed a hierarchical bi-factor structure among Spanish dental students. Using the questionnaire as a uni-dimensional scale may be useful in perceived stress level discrimination, while the sub-factors could help us to refine perceived stress analysis and improve therapeutic processes.

## Introduction

The training to become a health professional can create psychological distress and symptoms of burnout, which may have adverse consequences for one’s personal and professional life [1]–[5]. Dentists experience high levels of work-related stress, which begins during professional training [6]. Furthermore, the prevalence of burnout among dentistry students is high, with a significant relation found between the syndrome and a student’s academic performance, use of medication and thoughts of dropping out of the course [6]. Dental students complain of exhaustion resulting from the high levels of anxiety generated by exams, limited free time available for relaxation and the stress associated with having to adapt to the requirements of clinical practice [7]–[9]. As a result, some new dentistry graduates exhibit alarmingly high levels of burnout [10].

‘Stress’ occurs when environmental demands overwhelm individuals’ resources and threaten their personal well-being. It has been defined as the result of a relationship with the environment that individuals appraise as significant for their well-being and in which the demands exceed the available coping resources [11]. ‘Burnout’ is a response to the failure to cope adequately with chronic occupational stress and is an attempt to adapt to or to protect oneself from it [12]. This syndrome has classically been characterized by a state of exhaustion, cynicism and inefficacy [13]. ‘Exhaustion’ is the feeling of not being able to offer any more of oneself; ‘cynicism’ represents a distant attitude towards work, those served by it and other colleagues; and ‘inefficacy’ is the feeling of not performing tasks adequately or being incompetent.

On the other hand, ‘resilience’ has been characterized as a dynamic and flexible process of adaptation to life changes that could serve as a protective factor against psychological distress and mental disorders [14], [15]. It is the amount of personal strength, energy and motivation that enables an individual to cope with and recover from stress and to flourish when faced with adversity. Most of the proposed models to improve well-being in students and health professionals aim to enhance strategies for coping with stress using educational and environmental support as well as cognitive exercises that strengthen resilience skills [16], [17].

From the early stages of their university studies, dental students show concern for the stress produced by their experiences with clinical practice. They develop intense and long-term interactions with clients and patients, which is characteristic of careers with high levels of psychological distress and burnout [18]. Experiencing burnout over a prolonged period is associated with adverse emotional consequences, such as anxiety and depression, and it can also negatively affect the quality of patient care [19]–[24]. As a result, being able to measure the stress process in dental students seems to be essential if we want to evaluate their well-being along with their period of professional training.

The ‘Perceived Stress Questionnaire’ (PSQ) is a 30-item instrument that was developed by clinicians to quantify perceived stress [25]. It is one of the most used instruments that measure the stress process in psychosomatic research and has been associated with somatic complaints. In addition, it has demonstrated good predictive values in stress-related diseases [26]–[28]. The PSQ permits the subjective experience of perceived stressful situations and stress reactions to be assessed, emphasizing cognitive perceptions more than emotional states or specific life events. The general form of the instruction asks questions related to ‘the last two years’ and the recent form asks about situations taking place ‘during the last month’, potentially addressing chronic and acute relationships with stressful events and activities. It contains both positively and negatively formulated items in order to reduce acquiescent bias. Each item is answered using a four-point Likert-type scale, ranging from 1 (‘almost never’) to 4 (‘almost always’). Higher scores indicate more severe perceived stress. Originally designed in English, this instrument has been translated to Italian, German and Spanish, and validated in populations of psychiatric inpatients and outpatients, nursing students, health workers, psychosomatic patients and health adults [25], [27], [28].

One key element of this questionnaire is that it includes different groups of stressful experiences, such as harassment, overload, irritability, lack of joy, fatigue, worries and tension, which were proposed in its original version. However, this structure was not replicated. The study by Sanz-Carrillo [27] revealed the dimensions of social acceptance, load, irritability, energy, fear and self-realization; while the Fliege’s study [28] observed worries, tension, lack of joy and demands, differentiating the first three as internal stress reactions, while demands was classified as external stressors. So far, contemplating a general factor seems to be the most reasonable solution for its use. The original author proposed a linear algorithm ranging from 0 to 1 [25].

The previous inconsistencies may be due to differences among the samples that were used, which is in line with the idea that validating instruments in a specific population of interest is the most advisable option [29]. Nevertheless, the principal component analysis used as factor extraction method from r correlation matrices, along with the Kaiser role for the number of factors to retain, do not seem to be the best option to estimate factorial models using psychological variables as mentioned, and they are too often used out of force of habit [30].

Therefore, the goals of the present study were to evaluate the factor structure of the PSQ with dental students, together with its internal consistency model and its interrelatedness with the constructs of burnout, anxiety, depression and resilience. As a hypothesis, we expected high and significant correlations between all constructs.

## Methods

### Study Design

We used a cross-sectional design by means of the application of a self-assessment survey.

### Setting, Sample and Ethics Statement

The population consisted of Spanish dental students enrolled in Huesca (N_H_ = 136) and Santiago de Compostela (N_S_ = 242), during the 2010–11 academic year. An 83.1% response rate (RR) to the surveys, which were sent to all prospective participants, resulted in a sample of n = 314 participants. The students did not receive any financial or credit compensation for participating in the study. No differences were found in RR based on sex (males = 81.4% vs. females = 83.8%; p = 0.576), campus (Huesca = 87.5% vs. Santiago de Compostela = 80.6%; p = 0.085) or age (participants Mn = 22.05; SD = 3.57 vs. non-participants Mn = 22.34; SD = 3.83; p = 0.551).

A clinical psychologist trained two research assistants to administer the questionnaires as a battery in a paper-and-pencil format. The first page of the protocol identified the objectives of the study, participants, potential benefits and risks and the confidentiality of the data treatment. Each participant provided written informed consent before the commencement of the survey. The research assistants administered the survey in May 2011, two weeks before the period of final exams. After completion, the questionnaires were collected and kept in a sealed envelope to ensure the participants’ anonymity. The Ethical Review Board of Aragon, Spain, approved the study protocol. This study followed Helsinki Convention norms and later modifications, the Declaration of Madrid of the World Psychiatric Association and the Uniform Requirements for manuscripts submitted to Bio-medical journals.

### Measures

#### Socio-demographics

Data were collected on age, gender, stable relationship (‘yes’, ‘no’), children (‘yes’, ‘no’), campus (‘Huesca’, ‘Santiago’), distance from family home (km), residence (‘with parents’, ‘dormitory’, ‘shared flat’, ‘private flat’), scholarship (‘yes’, ‘no’), parental support perceived (‘insufficient’, ‘good’, ‘very good’), weekly study hours, failed subjects over the previous period (‘none’, ‘one’, ‘two or more’), job (‘yes’, ‘no’) and year of study (‘first’, ‘second’, ‘third’, ‘fourth’, ‘fifth’).

#### Perceived Stress Questionnaire (PSQ)

The Spanish version of PSQ (described above) was used, specifically its “recent form” addressing the previous 30 days [25], [27].

#### Goldberg Anxiety and Depression Scale (GADS)

The participants completed the Spanish version of the GADS [31], [32], which consists of a 9-item subscale that assesses symptoms of anxiety and a 9-item subscale that assesses symptoms of depression. The participants respond with either ‘yes’ or ‘no’, with one point scored for each positive response. A greater number of positive responses is associated with a greater likelihood of suffering from anxiety or depression. Each subscale provides a measure of the associated mental disorder. The convergence validity of the GADS has demonstrated adequate values of sensitivity and specificity [32]–[34]. We used this short and friendly scale because it is recommended for the screening of anxiety and depression in large sample studies in the general population [31]–[32].

#### Connor-Davidson Resilience Scale (10-item CD-RISC)

The participants also completed the Spanish version of the 10-item CD-RISC [35]–[36]. This scale is a self-report instrument that measures resilience on a 5-point Likert scale with responses that range from 0 (‘not at all’) to 4 (‘almost always’). The final scores are obtained by summing the response to each of the items, with higher values indicating higher levels of resilience. The validity and internal consistency of the scale were adequate and positively related to variables such as sleep quality and mental health [35], [36].

#### Maslach Burnout Inventory-Student Survey (MBI-SS)

Subjects were given the MBI-SS in its validated Spanish version for students [13], [37]. This questionnaire is the ‘golden rule’ for the evaluation of burnout, and this adaptation consists of 15 items grouped into the three dimensions: five items corresponded to exhaustion, four to cynicism and six to efficacy. Responses are arranged in a 7-point Likert scale, scored from 0 (‘never’) to 6 (‘always’). The scores of each dimension are obtained by adding up the responses to the corresponding items, with higher values indicating higher levels of each one. The questionnaire dimensions present an adequate structure and internal consistency [37].

### Data Analysis

SPSS v19.0, FACTOR v9.02 and AMOS v7.0 statistical packages were used to conduct the analysis.

#### Descriptives

Means, standard deviations, skewness, kurtosis and Mardia’s coefficients [38] were calculated to evaluate the performance of the PSQ items.

#### Dimensionality

Polychoric correlation is advised for factorial analysis (FA) when the distributions of ordinal items are asymmetric, with excess of kurtosis or with high item-rest coefficients [39]. Thus, a polychoric correlation matrix was estimated with regard to the PSQ items. We used parallel analysis (PA) [40] to identify the number of factors to include in the factorial solution, by replacing the raw data method [41] with optimal implementation based on minimum rank factor analysis [42], generating 500 random correlation matrices from permutation of the raw data. With this analysis, a factor is considered significant if the associated eigen-value is greater than that corresponding to 95th percentile of the distribution of eigen-values derived from a random dataset. PA is considered the best available solution to decide the number-of-factors-to-retain [43], [44]. We had previously verified the adequacy of the matrix, assessing the determinant, the KMO index and Barlett’s test of sphericity [45].

Unweighted Least Squares (ULS) was the method used for factor extraction [46]. The ULS procedure does not provide inferential estimations for assessing model data fit based on the χ^2^ distribution, but it has important advantages: it does not require any distributional assumptions; it is quite robust and usually converges because of its efficiency in terms of computation; and it tends to provide less biased estimates of the true parameter values [47]. Additionally, ULS is an appropriate choice for the case of not excessively large samples and shows good performance when working with polychoric matrices. In fact, ULS is consistent with the underlying variables approach from the Item Response Theory (IRT); it tends to supply accurate estimates even when models are large; and it provides better estimates than far more complex procedures [48]–[50]. The rotation method used was Promax, with a parameter of k = 4.00, given the correlated solution expected and using Raw Varimax as clever rotation start. We used Bentler’s simplicity index (S) and the loading simplicity index (LS) to evaluate factorial simplicity [51], [52].

Although we used unrestricted item factor analysis at this stage, we actually propose a second order factor solution, as a perceived stress general factor (G), by hierarchical factor solution [53]. This specification, which is multidimensional but is able to reflect the essential uni-dimensionality of the data, prescribes a factor which reflects what is common to all of the items, working as a multidimensional semi-confirmatory factorial analysis [54]. The factor weights (w) and the % of variance explained in each item by means of communality values (h^2^) were calculated. IRT parameterization by multidimensional normal-ogive graded response model (derived from the assumption of normally distributed measurement error), showed us the pattern of item discriminations in each dimension [55]. The belonging factor was determined by means of the IRT discrimination (a_n_), with those items with poor values being dismissed. Specifically, those items with a_n_ values <0.65 were discarded, as well as those with a_n_ values >0.65 in all of the latent dimensions at the same time [56].

We examined the fit of the proposed PSQ model by CFA, applying ULS from a polychoric matrix, for the reasons stated above. From a general perspective, we used the goodness-of-fit index (GFI), the adjusted goodness-of-fit index (AGFI), the root mean square of the standardized residuals (RMSR), the normed-fit-index (NFI) and Bollen’s relative-fit-index (RFI). GFI and AGFI refer to explained variance and values >0.90 are considered *acceptable* [57]. SRMR is the standardized difference between the observed and the predicted covariance, indicating a *good* fit for values <0.08 [58]. NFI measures the proportional reduction in the adjustment function when going from null to the proposed model and is considered *acceptable* when >0.90 [59]. RFI takes into account the discrepancy for the model evaluated and for the baseline model, and it is very *good* close to 1 [60]. All of them are perfectly valid for the ULS procedure. Taken together, they provide a reliable evaluation of the solution and additional information regarding absolute and incremental model fit. From an analytical perspective, standardized factor saturations and the explained variance were also considered.

#### Reliability

We examined the internal consistency of the scales and sub-escales using congeneric, tau-equivalent and parallel models [61]. The congeneric model is the least restrictive and assumes that each individual item measures the same latent variable, with possibly different scales, degrees of precision and magnitude of error. The tau-equivalent model implies that individual items measure the same latent variable on the same scale and with the same degree of precision, but with possibly different degrees of error. The parallel model is the most restrictive measurement model; it assumes that all items must measure the same latent variable on the same scale, with the same degree of precision and with the same amount of error. We finally chose the model that fitted better with the data, applying the ULS method and establishing comparisons. The reliability value was estimated by squaring the implied correlation between the composite latent true variable and the composite observed variable, to arrive at the percentage of the total observed variance that was accounted for by the true variable [62]. Mean inter-item polychoric correlations, item-rest and mean item-rest correlations were also used, as well as the mean Spearman’s R coefficients between the items over the belonging factor, calculated according to the Bayes ‘Expected A Posteriori’ (EAP) [63].

#### Convergence

Participant’s scores on PSQ factors calculated by EAP were also used in order to evaluate the degree of association between them and regarding the other constructs by means of Spearman’s R coefficients.

## Results

In order to adhere to standards for data availability, the authors state that all materials used to produce the results in this paper will be made available upon request. This includes [64]: 1.- The list of documents and data files that are needed in order for replication to be possible, 2.- A detailed list of what will be provided by the authors, and 3.- What steps, and in what sequence, the interested researchers need to take in order for this data to be made available. In addition, authors will post these materials on the group’s website [65].

### Participants

Table 1 displays the general characteristics of participants. They comprised adults of European ethnicity between the ages of 18 and 41 (Mn = 22.05; SD = 3.57), 70.70% of whom were women. Compared to students in Santiago de Compostela, students in Huesca lived further away from the family home (χ^2^ = 72.53; df = 2; p<0.001), were more likely to live in shared flats (χ^2^ = 14.79; df = 3; p = 0.002), were less likely to have received a scholarship (χ^2^ = 6.66; df = 1; p = 0.010) and failed a higher percentage of subjects over the previous exam period (χ^2^ = 7.33; df = 2; p = 0.026). Students in both Santiago and Huesca were similar with regard to the rest of the socio-demographic and occupational variables.

**Table 1:** Characteristics of the participants (n = 314).

Age, Md (SD)	22.05 (3.75)
Sex, females (%)	222 (70.7)
Stable relationship, no (%)	158 (50.5)
Children, none (%)	300 (95.5)
*Distance from family home (%)*	
<75 Km	110 (35.0)
75–150 Km	103 (32.8)
>150 Km	101 (32.2)
*Place of residence (%)*	
with parents	38 (12.1)
dormitory	51 (16.2)
shared flat	183 (58.3)
private flat	42 (13.4)
Scholarship, no (%)	199 (63.4)
Campus, Santiago (%)	195 (62.1)
*Family support (%)*	
insufficient	20 (6.4)
Good	74 (23.6)
very good	220 (70.0)
Weekly studying hours, Md (SD)	37.27 (17.52)
*Failed subjects (%)*	
None	212 (67.9)
One	78 (24.6)
two or more	24 (7.5)
Job, no (%)	266 (84.7)
*Year of study (%)*	
First	62 (19.8)
second	63 (20.0)
third	60 (19.1)
fourth	69 (22.0)
fifth	60 (19.1)

Md = Mean; SD = Standard Deviation; Number and percentage (%).

### Descriptives

Descriptive statistics for all the PSQ items can be seen in Table 2. Some items presented skewness values >1.00, such as ‘you feel lonely or isolated’ (item n° 5) and ‘you find yourself in situations of conflict’ (n° 6). Otherwise, some items showed kurtosis values <−1.00, as ‘you feel calm’ (n° 10 reversed), ‘you are under pressure from other people’ (n° 19), ‘you are afraid for the future’ (n° 22) and ‘you feel under pressure from deadlines’ (item n° 30). Mardia’s multivariate skewness and kurtosis coefficients were 132.70 (p = 1.00) and 1,040.71 (p<0.001), respectively.

**Table 2:** Descriptives, factorial solution, communalities and IRT parameterization of the PSQ items.

Items	Md	SD	skew	kurt	r_i(t−i)_	G	F_1_	F_2_	h^2^	a_1_	a_2_
1. You feel rested (r)	3.06	0.90	−0.69	−0.33	0.42	0.52	0.09	0.48	0.31	0.11	0.58
2. You feel that too many demands are being made on you *	2.65	0.94	−0.01	−0.94	0.52	0.60	−0.22	0.83	0.47	−0.31	1.14
3. You are irritable or grouchy	2.05	0.84	0.53	−0.24	0.58	0.65	0.37	0.40	0.50	0.52	0.56
4. You have too many things to do *	3.11	0.86	−0.57	−0.59	0.53	0.65	−0.30	0.95	0.58	−0.46	1.47
5. You feel lonely or isolated *	1.56	0.81	1.35	1.05	0.41	0.44	0.61	−0.04	0.35	0.76	−0.04
6. You find yourself in situations of conflict	1.66	0.83	1.12	0.50	0.43	0.40	0.44	0.07	0.24	0.50	0.08
7. You feel you’re doing things you really like (r) *	1.72	0.77	0.81	0.04	0.22	0.27	0.67	−0.26	0.27	0.78	−0.30
8. You feel tired *	2.83	0.90	−0.17	−0.93	0.56	0.69	0.11	0.65	0.54	0.17	0.97
9. You fear you may not manage to attain your goals *	2.34	0.95	0.32	−0.79	0.51	0.59	0.54	0.19	0.47	0.74	0.26
10. You feel calm (r) *	2.58	1.01	−0.13	−1.05	0.52	0.65	0.52	0.28	0.55	0.76	0.41
11. You have too many decisions to make *	2.59	0.80	0.15	−0.53	0.42	0.41	−0.32	0.71	0.29	−0.38	0.84
12. You feel frustrated *	1.80	0.83	0.86	0.19	0.49	0.58	0.77	−0.01	0.58	1.18	−0.02
13. You are full of energy (r) *	2.63	0.91	−0.19	−0.74	0.41	0.56	0.68	0.04	0.51	0.97	0.06
14. You feel tense *	2.38	0.91	0.21	−0.74	0.67	0.73	0.34	0.51	0.62	0.55	0.82
15. Your problems seem to be piling up	2.18	0.95	0.40	−0.75	0.70	0.75	0.42	0.46	0.67	0.73	0.79
16. You feel you’re in a hurry *	2.73	0.94	−0.23	−0.85	0.59	0.69	−0.09	0.82	0.57	−0.14	1.25
17. You feel safe and protected (r) *	2.29	0.91	<0.01	−0.95	0.40	0.48	0.70	−0.06	0.44	0.94	−0.08
18. You have many worries *	2.73	0.93	−0.14	−0.92	0.65	0.76	0.06	0.78	0.67	0.10	1.34
19. You are under pressure from other people	2.10	1.03	0.45	−1.02	0.58	0.63	0.38	0.36	0.47	0.52	0.50
20. You feel discouraged *	1.92	0.84	0.76	0.11	0.53	0.63	0.82	<0.01	0.68	1.45	<0.01
21. You enjoy yourself (r) *	2.12	0.95	0.38	−0.85	0.39	0.52	0.72	−0.03	0.49	1.00	−0.04
22. You are afraid for the future	2.40	1.02	0.14	−1.07	0.35	0.38	0.38	0.10	0.21	0.43	0.11
23. You feel you’re doing things because you have to (…) *	1.98	0.89	0.63	−0.34	0.51	0.50	0.49	0.13	0.35	0.66	0.16
24. You feel criticized or judged *	1.82	0.86	0.78	−0.22	0.48	0.50	0.62	0.03	0.41	0.81	0.04
25. You are light hearted (r) *	1.97	0.80	0.44	−0.38	0.42	0.53	0.94	−0.21	0.65	1.59	−0.35
26. You feel mentally exhausted *	2.52	0.96	0.10	−0.94	0.64	0.75	0.25	0.61	0.64	0.42	1.01
27. You have trouble relaxing *	2.27	1.03	0.37	−0.97	0.60	0.70	0.35	0.47	0.58	0.55	0.72
28. You feel loaded down with responsibility *	2.53	0.94	0.09	−0.90	0.67	0.77	0.17	0.69	0.67	0.29	1.21
29. You have enough time for yourself (r) *	2.97	0.89	−0.52	−0.52	0.47	0.61	0.15	0.53	0.42	0.20	0.70
30. You feel under pressure from deadlines *	2.80	0.95	−0.19	−1.01	0.58	0.67	0.03	0.70	0.52	0.04	1.01

**r_i(t–i_** = item-rest. G = second-order factor. F_1_ & F_2_ =  first-order factors. h^2^ =  communality. a_1_ & a_2_ =  IRT discrimination. r = reversed. ^*^conserved for later analyses.

### Dimensionality

The polychoric matrix of the PSQ items revealed that 78.6% of coefficients out of the diagonal were ≥0.30. The determinant was <0.001. KMO test had a value of 0.95 and Bartlett’s statistic was 4,780.50 (df = 435; p<0.001). The PA identified a two-factor structure, explaining 54.9% of the variance [(λ_1_ = 12.23; 46.7% variance of the real data; 7.5% variance explained over the P_95_ of the random samples); (λ_2_ = 2.39; 8.2% variance of the real data; 6.9% variance explained over the P_95_ of the random samples)]. The hierarchical solution by two first order factors (F_1_ and F_2_) and one second-order factor (G) exhibited good simplicity indices, such as S = 0.98 (P_99_) and LS = 0.45 (P_99_).

Table 2 shows the weights over G, the rotated loading matrix over F_1_ and F_2_ and the h^2^ values. All the items loaded ≥0.40 in G, except ‘you feel you are doing things you really like’ (n° 7 reversed; w_g_ = 0.27). F_1_ presented topics related to ‘frustration’, such as ‘you are light-hearted’ (n° 25 reversed; w_1_ = 0.94), ‘you feel discouraged’ (n° 20; w_1_ = 0.82) or ‘you feel frustrated’ (n° 12; w_1_ = 0.72). F_2_ exhibited themes associated with ‘tenseness’, such as ‘you have too many things to do’ (n° 4; w_2_ = 0.95), ‘you feel that too many demands are being made on you’ (n° 2; w_2_ = 0.83), or ‘you feel you are in a hurry’ (n° 16; w_2_ = 0.82). In general, h^2^ values were high, with an average of 0.49. Table 2 shows the PSQ items in terms of IRT discrimination. Some items did not present sufficient values, such as ‘you feel rested’ (n° 1 reversed; a_1_ = 0.11; a_2_ = 0.58), ‘you are irritable or grouchy’ (n° 3; a_1_ = 0.52; a_2_ = 0.56), and also items n° 6 (a_1_ = 0.50; a_2_ = 0.08), n° 19 (a_1_ = 0.52; a_2_ = 0.50) and n° 22 (a_1_ = 0.43; a_2_ = 0.11), already mentioned. Additionally, ‘your problems seem to be piling up’ (n° 15; a_1_ = 0.73; a_2_ = 0.79) presented high values in both factors, so it was also dismissed.

**Figure 1:**
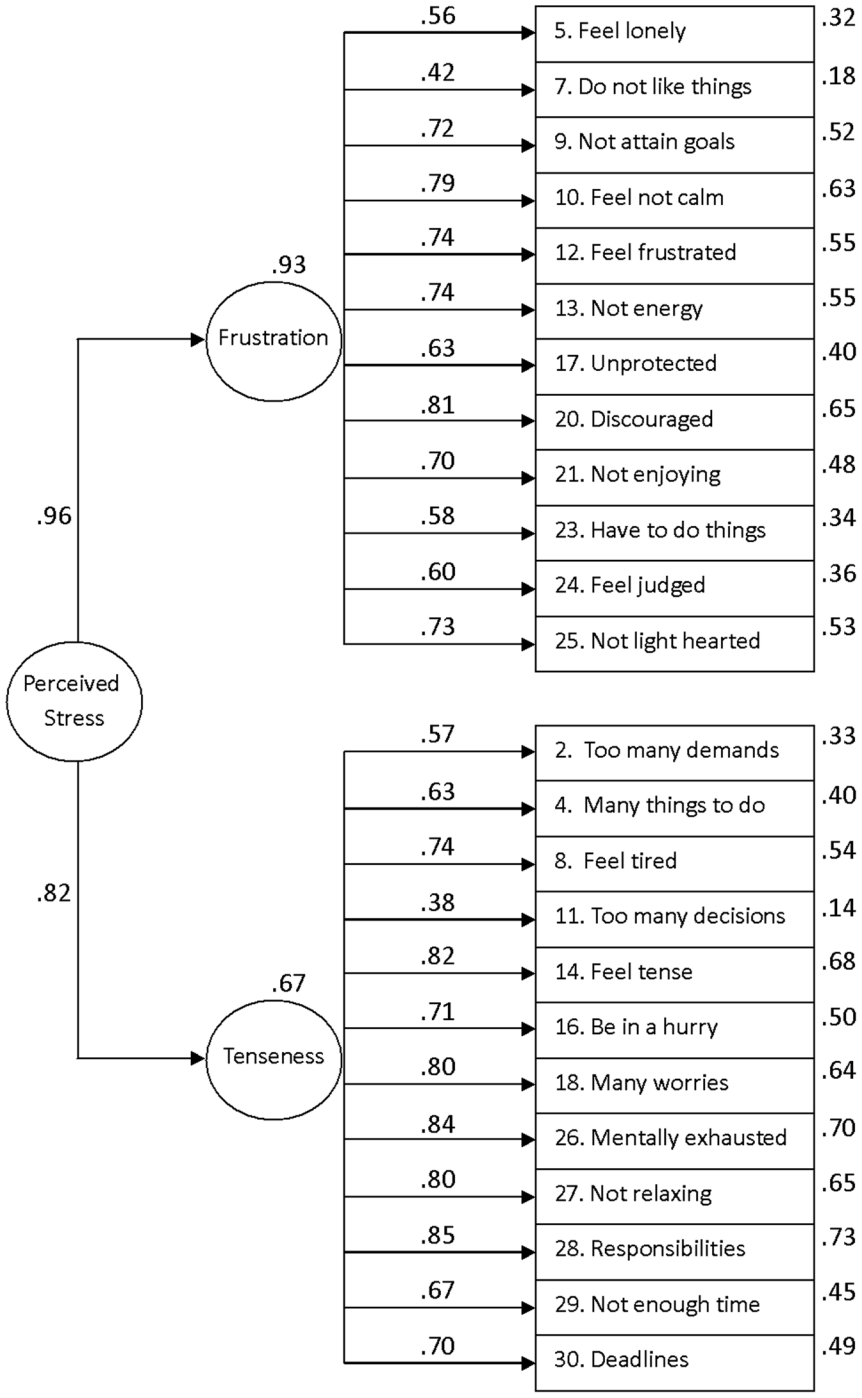
Construct validity of the PSQ hierarchical bi-factor structure. The circles represent latent constructs and the rectangles are observable variables. The factor weightings are over the one-way arrows and the percentage of explained variance for each observable variable over the boxes (standardized estimates).

Figure 1 shows the PSQ hierarchical bi-factor structure using CFA from an analytical and standardized point of view. The two first order factors turned out to be highly influenced by G, with loadings over F_1_ = 0.96 and F_2_ = 0.82. The item loadings with regard to their respective latent factor were high (F_1_ and F_2_ ranges  = 0.42 to 0.81 and  = 0.38 to 0.85, respectively). In a general sense, the PSQ hierarchical bi-factor structure presented adequate fit indices without using correlations between the error terms (GFI = 0.98; RSMR = 0.06; AGFI = 0.98; NFI = 0.98; RFI = 0.98).

### Reliability

Table 3 shows the reliability models tested for the PSQ hierarchical bi-factor structure. The indices fitted best with the congeneric model in all of the latent factors. Based on the congeneric model, the estimates of reliability obtained for G were 0.95; with 0.91 for F_1_ and 0.93 for F_2_. The mean inter-item polychoric correlation for the twenty-four selected PSQ items was 0.42. Item-rest values were positive and high, with an average of 0.59. All the items were highly and positively correlated to the belonging factor calculated by EAP, with an average of 0.65 over F_1_ and 0.70 over F_2_.

**Table 3:** Internal consistency models for the PSQ hierarchical bi-factor structure.

Factors/Models		R	GFI	AGFI	RSMR	NFI	RFI
**Perceived Stress**							
Congeneric		0.95	0.97	0.97	0.07	0.97	0.96
Tau-equivalent		0.95	0.90	0.89	0.15	0.88	0.88
Parallel		0.95	0.89	0.90	0.13	0.87	0.88
**Frustration**							
Congeneric		0.91	0.99	0.98	0.05	0.98	0.98
Tau-equivalent		0.91	0.95	0.94	0.11	0.93	0.93
Parallel		0.91	0.95	0.94	0.10	0.92	0.93
**Tenseness**							
Congeneric		0.93	0.99	0.99	0.05	0.99	0.99
Tau-equivalent		0.93	0.95	0.94	0.12	0.94	0.94
Parallel		0.93	0.95	0.94	0.10	0.93	0.94

R = Reliability; GFI = Goodness of Fit Index; RSMR = Root Mean Square of the Standardized Residuals; AGFI = Adjusted Goodness of Fit Index; NFI = Normed Fit Index; RFI = Relative Fit Index.

### Convergence

Table 4 shows the convergence values for the PSQ hierarchical bi-factor structure scores calculated by EAP. F_1_ and F_2_ presented a correlation of r = 0.62 and both had high associations with regard to anxiety, depression and exhaustion. However, F_1_ presented higher values with cynicism, positively, and with resilience and efficacy, negatively.

**Table 4:** Convergence values for the PSQ hierarchical bi-factor structure.

	rg	Md	SD	1	2	3	4	5	6	7	8
**1. Perceived Stress**	0–1	0.45	0.19								
**2. Frustration**	0–1	0.34	0.20	0.88*							
**3. Tenseness**	0–1	0.56	0.22	0.86*	0.62*						
**4. Anxiety**	0–9	5.30	2.65	0.60*	0.50*	0.56*					
**5. Depression**	0–9	3.39	2.31	0.67*	0.64*	0.56*	0.63*				
**6. Resilience**	0–40	27.81	6.74	−0.48*	−0.60*	−0.28*	−0.21*	−0.42*			
**7. Exhaustion**	0–30	13.49	7.49	0.76*	0.64*	0.70*	0.53*	0.66*	−0.34*		
**8. Cynicism**	0–24	5.57	4.74	0.43*	0.48*	0.26*	0.13†	0.40*	−0.32*	0.45*	
**9. Efficacy**	0–36	24.85	5.62	−0.28*	−0.43*	−0.08	−0.05	−0.21*	0.54*	−0.16‡	−0.38*

rg = range. Md = mean. SD = standard deviation. Perceived Stress (G), Frustration (F_1_) and Tenseness (F_2_) from PSQ. Anxiety and Depression from GADS; Resilience from 10-item CD-RISC; Exhaustion, Cynicism and Efficacy from MBI-SS. PSQ descriptives were calculated as: (raw score-24)/72. Frustration and Tenseness descriptives were calculated as: (raw score-12)/36. Convergence values are Spearman’s R correlations (PSQ factors calculated according to the Bayes ‘Expected A Posteriori’).

* p<0.001.

‡ p<0.01.

† p<0.05.

## Discussion

Despite the fact that perceived stress has been evaluated among dental students [9], as far as we are aware, this is the first factorial study of the PSQ among dental students. It is also the first study to examine its internal consistency model as well as possible interrelatedness with burnout, anxiety, depression and resilience. Other studies have evaluated its structure in other samples [27], [28], but the methods used did not respect the true ordinal nature of the variables, as we have. Our results provide evidence of a clear two-factor structure of the PSQ in dental students (‘frustration’ and ‘tenseness’), while it was also possible to use a single general factor (‘perceived stress’). Overall, the questionnaire had good psychometric properties, with adequate reliability and good convergence values, although it was advisable to discard some non-discriminative items. We found that the congeneric model was the optimal model to measure its internal consistency. Interestingly, the results revealed different relationship patterns between the perceived stress factors and the other constructs.

The main strength of the present study is that generalizability was enhanced because it was conducted using an high-stress-risk sample [4], [7]–[9], from two different universities in two Spanish regions, and these groups exhibited similar response rate. It is interesting to highlight the fact that the study was carried out during the period of final exams, a well-known source of distress, which may make the results more relevant [66]. The response rate was high and the participants did not differ significantly from non-participants with regard to age, gender or years of study. Moreover, an independent researcher supervised the data transcription process to control for errors, and the analysis method respected the true nature of the variables used. The main limitation of the study was the use of a cross-sectional design because it did not permit the analysis of causal hypotheses. Another limitation could proceed from the instruments used, because they are not the only questionnaires used to measure such constructs. The use of other questionnaires might have produced slightly different results.

The participants in the study were young adults. Most of the individuals were women who did not have children. The majority of students also did not receive financial assistance and were not employed. On average, the responses of the participants were not extreme, although the values for tenseness and anxiety were moderately high. Comparatively, the scores for frustration, burnout and depression were slightly lower. This findings can be understood by considering the pressure that the students experienced owing to the proximity of final exams.

One of the most salient findings of this study is the clear hierarchical bi-factor structure shown by the PSQ among Spanish dental students. In this population, high correlations were observed between one second-order factor and two first-order factors. The second order factor (G) refers to ‘perceived stress’ as a general factor, as used in the original proposal [25], [26] and in other studies [27], [28]. The first of the first-order factors (F_1_), which we referred to as ‘frustration’, mainly included items from the original ‘lack of joy’ and ‘worries’. The second of the first-order factors (F_2_), named ‘tenseness’, mainly included items from the original ‘tension’ and ‘overload’ [25]. In other words, F_1_ consisted of the stress perceived as lack of joy and worries, and F_2_ consisted of the external stressor of demands and the stress reaction of tension [28].

In general terms, the behaviour of the items was adequate, with high and positive item-rest values, although their distributions suggest a non-linear analysis, as was expected. All the items weighted strongly and positively in G factor. However, the IRT discrimination values advised us to reject some items because they were unable to differentiate adequately between F_1_ and F_2_. For these reasons, the 24 selected items were strong and positively weighted in the belonging factor and the model fit was very good. These items correlated highly with one another and internal consistency values were high, for G as well as for F_1_ and F_2_. Each item seemed to be measuring the corresponding latent variables, with possibly different degrees of precision and different amounts of error.

We found that G was significantly related to all the considered constructs, and therefore this finding supports the hypothesis that burnout syndrome could mediate the link between perceived stress and the occurrence of emotional disorders [67], [68], although it is well known that there are many others sources of stress, different from those related to work or workers in training. In other words, occupational stress could be an important risk factor for the development of anxious and depressive symptoms in dental students through burnout syndrome [69]–[71]. Thus, dealing with stress problems at an early stage of training may help to promote resilience and reduce burnout and mental health problems in the longer term [72]. Nevertheless, in order to reach a full understanding of the process, it will be necessary to continue with research until the hypothetical relationships put forward can be fully brought to light. It will be necessary to make use of designs that allow possible causal relationships to be explained, particularly if we are interested in developing effective lines of intervention, given the lack of these in a setting that is so in need of them.

In addition, the pattern of relationships observed between the PSQ first-order factors and the other constructs suggests that while both F_1_ and F_2_ seems to be strongly associated with exhaustion, anxiety and depression, F_1_ could be more positively related than F_2_ to cynicism, and more negatively related to efficacy and resilience. In general, resilience increases the feelings of satisfaction and commitment that promote the empowerment of and beneficial outcomes for students and even workers [73]–[77]. In accordance with the demands-resources model [78], people who suffer from burnout experience a progressive decline in commitment to their studies over time [79]. This is perhaps why resilience constitutes a coping reservoir that influences the long term functioning of university students [17,72). However, our results suggest that interventions focused on promoting resilience may be of benefit in overcoming frustration more than tenseness, while tenseness seems to be more important in this population. Combining resilience interventions with relaxation interventions could possibly be the best choice for dental students.

## Conclusions

The PSQ showed a hierarchical bi-factor structure among Spanish dental students. Using the questionnaire as a unidimensional scale may be useful for discerning perceived stress levels, while the two sub-factors could help us to refine the perceived stress analysis and improve therapeutic processes. On the other hand, certain important psychometric aspects of the PSQ with regard to dental students remain unknown and should be examined in the future: first, whether the PSQ hierarchical bi-factor structure varies across male and female students; and second, whether differences in translations of the PSQ to other languages may have introduces variations as regards its factor structure and item loadings. Therefore, the PSQ hierarchical bi-factor structure model should be replicated in a large and multi-national sample of dental students.
